# Infecciones en el trópico: retos para la investigación aplicada

**Published:** 2020-08-20

**Authors:** María Fernanda Yasnot, Ramón Gamarra, Clara B. Ocampo

**Affiliations:** 1 Junta Directiva, Asociación Colombiana de Parasitología y Medicina Tropical Asociación Colombiana de Parasitología y Medicina Tropical

Las infecciones causadas por microorganismos patógenos como virus, bacterias, protozoos o helmintos, que dependen de un huésped para su supervivencia, representan más del 25 % de las enfermedades a nivel global.

Estos agentes infecciosos se transmiten por contacto entre los huéspedes o por organismos vectores. En las regiones tropicales, la transmisión de los agentes infecciosos se ve favorecida por la ecología, el clima y el nivel de desarrollo social, lo que desemboca en serios problemas de salud pública.

Las enfermedades transmitidas por insectos vectores, como el dengue, la leishmaniasis, la tripanosomiasis y la malaria, y aquellas transmitidas por contacto, como las enfermedades diarreicas, la tuberculosis y las infestaciones helmínticas, siguen registrándose en los países en desarrollo como Colombia. En nuestro país, a pesar de los grandes esfuerzos y la inversión en las estrategias de prevención y control, persiste la influencia de la migración, el conflicto armado en algunas áreas rurales, la pobreza económica, los bajos niveles educativos de la población, la falta de condiciones higiénico-sanitarias, y los limitados recursos para la planeación y la toma de decisiones adecuadas y continuas por parte de los organismos responsables de la salud pública ^1^^,^^2^.

Estos factores, sumados a la gran movilidad en el mundo, favorecen la reemergencia y la emergencia de nuevos agentes patógenos y su diseminación global. Un ejemplo de ello es la actual pandemia de SARS- CoV-2, que se inició en diciembre de 2019 en Wuhan, China, y se ha dispersado a todos los países del mundo: el 1° de mayo de 2020 ya se habían confirmado 3’214.256 casos y 232.570 muertes ^3^. Otro ejemplo es el del virus del Ébola, que en el 2014 se presentó inicialmente en Sierra Leona, Liberia y Guinea y, posteriormente, se extendió a los países vecinos (Malí, Nigeria y Senegal) e, inclusive, a los Estados Unidos y España, causando más de 7.000 muertes en el mundo ^4^. Por otro lado, en Latinoamérica aparecieron los virus del chikungunya (2014) y el Zika (2015), transmitidos por mosquitos urbanos del género *Aedes*, que produjeron epidemias y afectaron a más del 80 % de la población en los diferentes países ^5^^-^^7^.

La rápida transmisión de las enfermedades infecciosas ha cimentado la conciencia sobre su potencial epidémico y pandémico, y el consecuente trastorno en poco tiempo del aparente equilibrio de las sociedades. Dichas experiencias han resaltado la necesidad de replantear las estrategias de contención, incluidos los cambios de conducta frente a estas enfermedades, las investigaciones que buscan disminuir su impacto mediante tratamientos, vacunas, biomarcadores de diagnóstico y pronóstico, nuevos dispositivos médicos, vigilancia epidemiológica con herramientas matemáticas y geográficas, así como los cambios en las políticas públicas.

Todo ello lleva a pensar que se necesita un sistema integrado de salud que permita la interacción entre los entes gubernamentales (nacionales y territoriales) del sector salud y los investigadores, con el fin de disminuir la brecha existente entre las decisiones que se toman y la implementación de los resultados de la investigación científica. El trabajo integrado permitiría establecer las estrategias prioritarias para reducir los riesgos en salud y manejar las situaciones epidémicas en el país.

En los últimos 10 años, muchos países, incluido Colombia, han aumentado las inversiones para la prevención y el control de las enfermedades tropicales. Asimismo, la Organización Mundial de la Salud (OMS) ha intensificado sus esfuerzos para contrarrestar su impacto mundial, permitiendo mejoras en la salud colectiva y el bienestar social. Sin embargo, aún persisten muchos problemas que deben ser abordados en las agendas anuales de los gobiernos y en los planes de desarrollo nacionales y departamentales. Además, es necesario promover la coordinación de todos los responsables del bienestar y la salud de las poblaciones y de los investigadores, con el fin de profundizar en los temas regionales prioritarios.

En el “Informe sobre la salud en el Mundo, 2013”, la OMS plantea como uno de los argumentos a favor de la inversión en investigación el hecho de que los países podrían aprovechar más eficazmente la oferta de ideas utilizando distintos métodos de investigación: las evaluaciones cuantitativas y cualitativas, los estudios observacionales y de casos y controles, los estudios de intervenciones, los estudios aleatorizados controlados, y las revisiones sistemáticas y metaanálisis, para que se traduzcan en productos y estrategias que aporten a los programas y ayuden a tomar decisiones en salud pública ^8^.

Teniendo en cuenta que el avance y la competitividad de las sociedades contemporáneas se sustenta en el conocimiento, el fortalecimiento de la educación de alta calidad y el desarrollo de las capacidades científicas y de innovación, en el 2011, Colombia aumentó su inversión en este campo y creó el Fondo de Ciencia, Tecnología e Innovación del Sistema General de Regalías con un aporte del 10 % de las regalías del país, destinado a promover la ciencia, la tecnología y la innovación. Esta inversión, unida a la reciente creación del Ministerio de Ciencia, Tecnología e Innovación, ha mejorado la capacidad instalada de algunos centros y grupos de investigación, la capacitación y formación superior de talento humano y la inversión en estudios con miras al fortalecimiento regional. Sin embargo, se hace necesaria la financiación sostenida que garantice que los proyectos de investigación no se vean interrumpidos ni sus resultados afectados a mediano y largo plazo.

Con dicha inversión y la acción de los programas departamentales enfocados en las enfermedades infecciosas tropicales, se ha logrado un avance en la interacción entre los entes gubernamentales y los investigadores. Sin embargo, es necesario fortalecer las capacidades institucionales para una respuesta adecuada, organizada y oportuna, así como establecer una estrategia basada en mecanismos de alerta temprana y de respuesta rápida que cuente con recursos humanos, laboratorios y canales de comunicación entre los laboratorios y los servicios de salud, para mejorar la vigilancia de los agentes infecciosos, los vectores y reservorios, los factores de riesgo, y los elementos ambientales y climáticos que favorecen la aparición de epidemias.

Es importante promover entre los grupos académicos el contacto con las sociedades científicas nacionales e internacionales que promueven la colaboración y las alianzas entre investigadores para el desarrollo de proyectos científicos que generen nuevo conocimiento y ofrezcan soluciones en las áreas de interés. Este es el caso de la Asociación Colombiana de Parasitología y Medicina Tropical que, desde su creación hace más de 30 años, ha perseverado en el propósito de fomentar el avance del conocimiento de la parasitología y la medicina tropical mediante actividades científicas, educación continua e investigación.
